# LST-EMG-Net: Long short-term transformer feature fusion network for sEMG gesture recognition

**DOI:** 10.3389/fnbot.2023.1127338

**Published:** 2023-02-28

**Authors:** Wenli Zhang, Tingsong Zhao, Jianyi Zhang, Yufei Wang

**Affiliations:** ^1^Faculty of Information Technology, Beijing University of Technology, Beijing, China; ^2^College of Art and Design, Beijing University of Technology, Beijing, China

**Keywords:** sEMG signals, gesture recognition, multi-scale features, multi-head attention, stroke rehabilitation, human-computer interaction

## Abstract

With the development of signal analysis technology and artificial intelligence, surface electromyography (sEMG) signal gesture recognition is widely used in rehabilitation therapy, human-computer interaction, and other fields. Deep learning has gradually become the mainstream technology for gesture recognition. It is necessary to consider the characteristics of the surface EMG signal when constructing the deep learning model. The surface electromyography signal is an information carrier that can reflect neuromuscular activity. Under the same circumstances, a longer signal segment contains more information about muscle activity, and a shorter segment contains less information about muscle activity. Thus, signals with longer segments are suitable for recognizing gestures that mobilize complex muscle activity, and signals with shorter segments are suitable for recognizing gestures that mobilize simple muscle activity. However, current deep learning models usually extract features from single-length signal segments. This can easily cause a mismatch between the amount of information in the features and the information needed to recognize gestures, which is not conducive to improving the accuracy and stability of recognition. Therefore, in this article, we develop a long short-term transformer feature fusion network (referred to as LST-EMG-Net) that considers the differences in the timing lengths of EMG segments required for the recognition of different gestures. LST-EMG-Net imports multichannel sEMG datasets into a long short-term encoder. The encoder extracts the sEMG signals’ long short-term features. Finally, we successfully fuse the features using a feature cross-attention module and output the gesture category. We evaluated LST-EMG-Net on multiple datasets based on sparse channels and high density. It reached 81.47, 88.24, and 98.95% accuracy on Ninapro DB2E2, DB5E3 partial gesture, and CapgMyo DB-c, respectively. Following the experiment, we demonstrated that LST-EMG-Net could increase the accuracy and stability of various gesture identification and recognition tasks better than existing networks.

## 1. Introduction

Surface electromyography signals are bioelectric signals generated during muscle contractions. sEMG signals can be collected non-invasively, safely, and easily, and sEMG can directly reflect the state of muscle activity. By analyzing the sEMG, gestures can be accurately recognized. sEMG-based gesture recognition methods have the advantages of being faster and more environmentally independent than vision-based gesture recognition methods (Oudah et al., 2020; Mujahid et al., 2021). Therefore, sEMG-based gesture recognition methods have strong application possibilities in sectors related to human-computer interfaces, including intelligent prosthetics (Cipriani et al., 2008), upper limb rehabilitation exoskeletons (Leonardis et al., 2015), robotic arm control (Wang et al., 2012), and others (Muri et al., 2013).

An sEMG-based gesture recognition framework generally consists of four parts: Signal preprocessing, signal segmentation, feature extraction, and gesture classification mechanisms (Parajuli et al., 2019). For traditional machine learning algorithms, the features used for classification are usually handcrafted by human experts. Therefore, the quality of the feature set selected by the experts directly determines the success or failure of the recognition task. Numerous gesture recognition studies have used traditional classifiers for manual features. For example, support vector machines (SVMs) (Alseed and Tasoglu, 2022; Briouza et al., 2022), k-nearest neighbors (KNN) (Baygin et al., 2022), linear discriminant analysis (LDA) (Narayan, 2021), hidden Markov models (HMMs) (Hu and Wang, 2020), and random forests (RF) (Xue et al., 2019; Jia et al., 2021) have made some progress. However, the accuracy and stability of traditional learning algorithms do not yet satisfy practical application requirements when applied to large-scale datasets consisting of larger numbers of hand gestures or wrist movements. Therefore, improving the accuracy and stability of hand gesture recognition is still a hot research topic.

In recent years, with the rapid development of artificial intelligence technology, deep learning has shown great potential in medical rehabilitation fields such as physiological signal processing (Rim et al., 2020; Al-Saegh et al., 2021) and medical image imaging (Karim et al., 2022; Laghari et al., 2022). In gesture recognition tasks based on surface EMG signals, deep learning methods can automatically learn the dependencies or intrinsic connections of the amplitude changes at each sampling point in surface EMG signals due to their deep network architectures. The dependencies and intrinsic connections can be considered the muscle activity features that indirectly express forearm muscle activity conditions, and gesture information can be obtained under this condition. The following research summarizes the feature extraction methods that have been developed under different model architectures for deep learning algorithms.

### 1.1. Related work

Deep learning models outperform traditional machine learning models, so many researchers use deep learning for gesture recognition. Convolutional neural networks (CNNs) (Atzori et al., 2016; Wei et al., 2019; Chen et al., 2020), recurrent neural networks (RNNs) (Vaswani et al., 2017; Hu et al., 2018; Xia et al., 2018), and transformer-based gesture identification approaches (Rahimian et al., 2021; Siddhad et al., 2022) are the current prevalent deep learning gesture recognition algorithms.

Researchers have conducted studies on CNN-based gesture recognition methods (Atzori et al., 2016; Wei et al., 2019; Chen et al., 2020). Atzori et al. (2016) first applied a CNN to an sEMG gesture recognition task using only a shallow network constructed from four convolutional layers. The accuracy was comparable to that of traditional machine learning gesture recognition methods. Wei et al. (2019) proposed a multistream convolutional neural network (MSCNN) with decomposition and fusion stages. The network learned the correlations between gesture muscles, and it was evaluated on three benchmark databases. The results showed that multistream CNNs outperformed simple CNNs and random forest classifiers, but the computational effort of the method increased multiplicatively with the number of myoelectric channels.

Some researchers have combined recurrent neural networks (RNNs) with CNNs, using CNNs for feature extraction and RNNs for modeling time dependencies (Vaswani et al., 2017; Hu et al., 2018; Xia et al., 2018). An RNN has all nodes connected in a chain-like manner, so it can handle short-term memorization tasks well. For example, Xia et al. (2018) proposed the RCNN, a single-branch deep structure with a CNN and RNN connected serially. The CNN extracts the myoelectric local spatial features, and the RNN saves the local spatial features and efficiently passes them to the next moment to update the model weights. This network has an advantage in learning complex motion features. However, the RCNN can have a sharp decrease in recognition accuracy over time compared to the CNN. Xia et al. (2018) tried to use large neural networks with more layers and parameters to improve the robustness of the model to time variations. Nevertheless, problems such as a heavy training time burden and system recognition delays are caused by the inability of RNNs to train in parallel. Hu et al. (2018) proposed an attention-based hybrid CNN-RNN model. The model uses a CNN to extract spatial feature maps of successive frames of sEMG signals and an RNN to further extract temporal features from the feature maps. The model was able to effectively extract the temporal correlation of each channel of sparse multichannel sEMG signals.

In recent years, after the transformer model (Vaswani et al., 2017) was proposed, it attracted attention in natural language processing and computer vision tasks. The transformer model entirely relies on self-attention, which can capture global dependencies in the input data to achieve parallel computation and improve computational efficiency. At the same time, self-attention can produce more interpretable models. For example, Siddhad et al. (2022) explored the effectiveness of transformer networks for the classification of raw EEG data. They used raw resting-state EEG data to classify people by age and gender, and the classification results showed that the transformer network was comparable in accuracy to state-of-the-art CNN and LSTM recognition with feature extraction. This proved that the transformer network has excellent feature extraction capability for time-series signals. Some researchers have already used transformers for hand gesture recognition (Rahimian et al., 2021; Montazerin et al., 2022). For example, Rahimian et al. (2021) used a vision-based transformer model architecture for the classification and recognition of upper limb gestures, and the recognition accuracy on Ninapro DB2 Exercise B for 17 gestures reached state-of-the-art performance at that time. Montazerin et al. (2022) also proposed a transformer framework based on ViT for high-density sEMG gesture recognition with 128 arrayed electrodes, which can solve the time burden problem of RNN structures that are not trained in parallel. However, current transformer-based gesture recognition networks only apply the image classification scheme to EMG recognition. The network structure is not designed according to the characteristics of gesture activity and sEMG signals.

In summary, gesture recognition is mainly implemented by deep learning methods at this stage. Among them, the transformer model has become a hot research topic because of its self-attention structure, which can extract sEMG signal temporal muscle activity features well and performs well in gesture recognition. However, current recognition methods still suffer from a mismatch between the amount of information contained in the extracted features and the amount of information required to recognize gestures when implementing multicategory gesture recognition. The reason for the mismatch problem is that there are differences in the stability exhibited in the sEMG signal due to the different muscle activity, muscle contraction changes, and muscle strength changes mobilized (Farago et al., 2022; Li et al., 2022). The sEMG signals of more complex gestures are less stable, and simpler gesture movements have better EMG signal stability. To recognize complex gestures from less stable EMG signals, the lengths of the feature extraction segments need to increase to yield a sufficient amount of information for recognition (Nazmi et al., 2017). However, most of the existing related works do not consider the characteristic that the lengths of EMG signals are different for different gestures. They all intercept fixed-length EMG signals for spatial and temporal feature extraction, which leads to a mismatch between the amount of feature information extracted by the designed models and the corresponding gestures and affects the accuracy and robustness of the gesture recognition framework.

### 1.2. Contributions

To address the above problems, it is necessary to propose a gesture recognition method to extract moderate feature information from EMG sample segments. Therefore, we propose a gesture recognition method based on LST-EMG-Net. It can extract long- and short-sequence features in sEMG windows and fuse them effectively to achieve high-accuracy recognition of complex and simple gestures. The method proposed in this article makes the following three contributions:

(1) To address the mismatch between the feature information and required information in a multicategory gesture recognition task, we propose a long short-term encoder and use the linear projection in the encoder to construct a long-term branch and a short-term branch. Then, each branch feature is extracted by a long- or short-term subencoder to achieve multiscale time feature extraction and solve the problem of redundant or insufficient feature extraction. To further improve the feature quality, we use sEMG channel attention to dynamically set the weights of each channel of the sEMG windows.
(2) We propose a cross-attention module for long- and short-term features from the encoder to fuse the long- and short-term features efficiently. This module uses an attention-based approach to cross-learn one branch’s classification token and another branch’s patch tokens in the feature. This module can effectively fuse the muscle activity information and enhance the efficiency of feature fusion due to its low computational effort. It finally achieves the goal of improving the accuracy of hand gesture recognition.
(3) To address the problem that individual sEMG signals are difficult to collect in large quantities, we propose a signal augmentation method based on sEMG windows. This method adopts random windows and sEMG time delays to augment the original sEMG windows and constructs a training dataset together with the original EMG timing windows. This method reduces the burden of data collection.

The remainder of the article is organized as follows. The dataset and the sEMG signal enhancement method utilized in this article are described in detail in Section “2. Materials and methods.” The framework of LST-EMG-Net, including the motivation of the study and the submodule structure, is presented in Section “3. The long short-term sEMG transformer feature fusion network framework.” The experimental environment of LST-EMG-Net and experimental results are presented in Section “4. Experiments and results.” Finally, the conclusions of this article are drawn in Section “5. Conclusion.”

## 2. Materials and methods

### 2.1. The datasets

We use two types of datasets, a sparse sEMG dataset and a high-density sEMG dataset, to evaluate our LST-EMG-Net. The sparse dataset includes Ninapro DB2 and DB5 (Atzori et al., 2012, 2014a,b; Gijsberts et al., 2014; Du et al., 2017; Pizzolato et al., 2017). The high-density dataset is the public CapgMyo dataset (Du et al., 2017).

**FIGURE 1:**
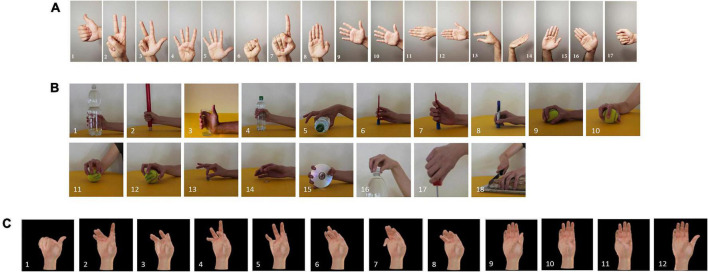
Types of gestures in the datasets used in this manuscript. **(A)** Ninapro DB2 exercise B dataset 17 gestures. **(B)** Ninapro DB5 exercise C dataset 18 gestures. **(C)** CapgMyo DB-c dataset 12 gestures.

Sparse sEMG dataset: We use 17 basic wrist movements and isotonic hand configurations from the DB2 Exercise B subdataset (as shown in Figure 1A). In the DB2 dataset, the muscular activities were measured using 12 active double-differential wireless electrodes from a Delsys Trigno Wireless EMG system at a sampling frequency of 2 kHz. The DB5 dataset uses 18 gestures from the Exercise C subdataset that fully mobilize muscle activity and facilitate muscle recovery training (as shown in Figure 1B). The DB5 dataset was taken from 10 healthy subjects. Its collection device was a pair of Thalmic Labs Myo (Myo) armbands. Each Myo had eight single-channel electrodes, each with a sampling rate of 200 Hz. The DB2 dataset and DB5 dataset collection rules were the same. Each gesture was repeated six times, each acquisition had a 5 s activity signal, and each acquisition interval was 3 s. The 1st, 3rd, 4th, and 6th repetitions of the gesture were used to construct the training set, and the 2nd and 5th repetitions were used to build the test set.

High-density sEMG dataset: We used 12 basic finger movements from the DB-C subdataset of CapgMyo (as shown in Figure 1C). The dataset was acquired at a sampling rate of 1,000 Hz with an array of 8 2 × 8 differential electrodes to capture the activity of the right peripheral forearm muscle groups. The CapgMyo dataset was obtained from 10 users who repeated several movements 10 times, each lasting 3 s, followed by 7 s of rest. Odd-numbered repetitions were used to construct the training set, and even-numbered repetitions were used to build the test set.

### 2.2. Preprocessing

Before performing the classification task, the sEMG signals were preprocessed. The sEMG signals were filtered from power line interference before signal acquisition due to the built-in 50 Hz trap filter in the sEMG sensor. We only used a blind source separation process called fast independent component analysis (Fast ICA) (Comon, 1992) on the raw signals to eliminate interchannel crosstalk. Then, Z Score standard normalization was used to process the sEMG signals after filtering the noise. Z Score normalization of a channel is shown in Equation 1.


(1) $$F\,\left(x_{t}\right)=\frac{x_{t}-\mu }{\sigma }$$

Where *x_t_* is the sEMG signal, μ is the mean value of the sEMG signal and σ is the standard deviation of the sEMG signal.

This article uses the sliding-window method with overlap to segment the normalized EMG signal to obtain the original EMG timing window. The length of the sliding window is set according to the related work of Scheme and Englehart (2011). It is noted that 300–800 ms is an acceptable latency. Considering the delay and computation volume, we set the window length of the Ninapro DB2 dataset as 300 ms, its window distance as 10 ms, the window length of the Ninapro DB5 dataset as 500 ms, its window length as 100 ms, the window length of the CapgMyo dataset as 60 ms and its window distance as 10 ms.

### 2.3. Signal augmentation based on sEMG windows (SA)

Due to the lack of *a priori* experience of the self-attention of the transformer network, such as inductive bias and translational invariance, the transformer model requires a larger dataset to reach convergence. However, most current recognition methods require the acquisition of individual sEMG signals to build recognition models, whereas the collection of sEMG data from a large number of individuals is difficult to achieve because of the high time cost and muscle fatigue. Therefore, we propose a signal augmentation method based on sEMG windows to solve the above problem. This method is used to obtain random windows and time-delay enhancement windows to increase the number of training samples.

First, the original sEMG signals are input into the random window selection module. This module randomly selects the start point of the window within each class of gesture action sequences and determines the end point based on the window length to obtain a random window for that type of gesture. This operation is repeated to obtain the random windows for all gesture actions.

**FIGURE 2:**
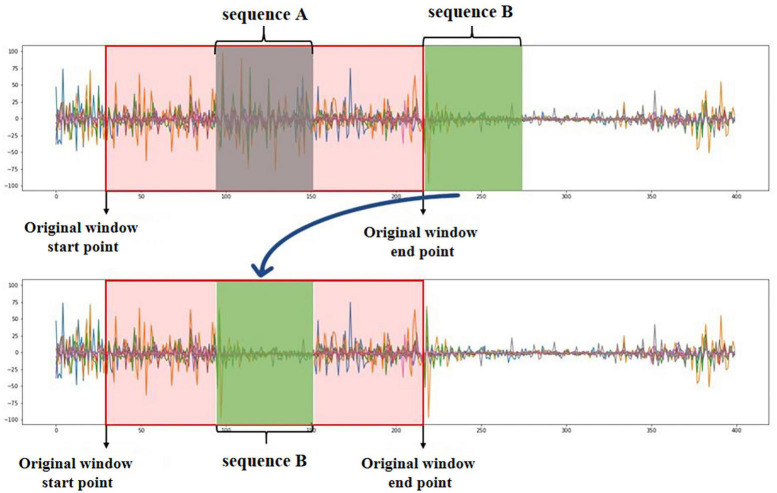
Schematic diagram of time-delay enhancement module.

Second, the transceiver delay and transmission interference of the acquisition device (Liu et al., 2016) make it inevitable that sEMG will miss some sample points, which impacts the sEMG recognition model’s robustness. Therefore, this step randomly selects a certain percentage of the original sEMG window to input to the time-delay enhancement module. This model selects sequence An in the original window randomly and selects sequence B at the next sampling moment (where the numbers of sampling points of sequence A and sequence B are the same); finally, sequence A is deleted, and the sampling points of sequence B are copied to the original sequence A sampling moment to obtain the time-delay enhancement windows, as shown in Figure 2.

The proposed signal augmentation method expands the training samples by doubling the number of sEMG windows. Take DB5 dataset subject ten as an example: we have 5,886 original EMG windows initially; then, we obtain 2,943 random windows by the random window selection module and 2,943 time-delay enhanced windows by the time-delay enhancement module. Finally, we obtain 11,772 windows.

## 3. The long short-term sEMG transformer feature fusion network framework

In the field of gesture recognition, we often use the fractal dimension to calculate the complexity and stability of the EMG signal (Naik and Nguyen, 2014). Gestures with low fractal dimensions are simple gestures whose signal stability is higher, and the electrode position, muscle contraction, and muscle force change more slowly in these types of gestures; they include single-finger flexion, multifinger flexion, and wrist translation (Namazi, 2019a). Gestures with high fractal dimensions are complex gestures with low signal smoothness. The electrode position, muscle contraction, and muscle force change more rapidly in these gestures, such as wrist-hand linkage and dynamic operations (e.g., grasping, pressing, and tapping). In addition, when the subject increases the force of the gesture, it also leads to an increase in the fractal dimension of the EMG signal (Menon et al., 2017; Namazi, 2019b), which in turn affects the stability of the EMG signal. Therefore, we need longer EMG sequence segments to extract high-quality features from signals with low stability when recognizing complex gestures; we need shorter EMG sequence segments to recognize simple gestures.

On the other hand, the transformer can capture longer dependent information in the temporal signal classification task due to its self-attention structure. However, current transformer networks are designed based on multihead attention; in this method, there is a lack of constraints between every pair of heads, which makes the output similar between network layers, eventually leading to the problem of attention collapse (Zhou et al., 2021) and affecting accuracy. Therefore, we propose LST-EMG-Net to extract the sEMG features of both long-term and short-term segments in the sEMG window to perform multitemporal feature extraction and feature fusion for various-complexity gesture recognition tasks. To further improve the gesture recognition effect, the network adds a transposition matrix between the heads of multihead attention to solve the attention collapse problem.

**FIGURE 3:**
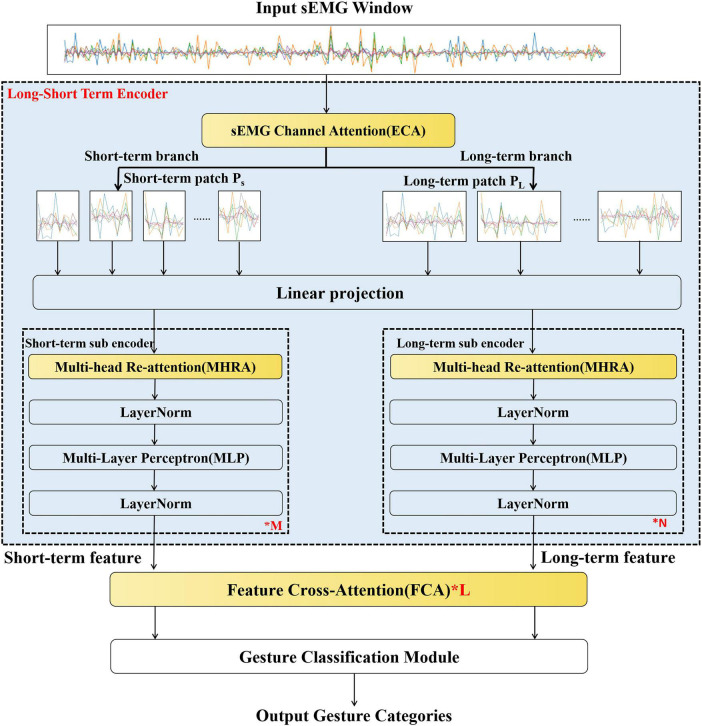
The proposed LST-EMG-Net structure. Among them, the sEmg channel attention, multi-head re-attention and the feature cross-attention module (yellow module) are the contribution points of this manuscript.

The overall structure of LST-EMG-Net is shown in Figure 3; it consists of three parts: the long short-term encoder, feature cross-attention module, and gesture classification module, of which the long short-term encoder module and the feature cross-attention module correspond to contributions 1 and 2 of this article, respectively.

Long short-term encoder: This module takes as input a set of multichannel sEMG window collections $D=\left\{\left(X_{i},y_{i}\right)\right\}\,\frac{m}{i=1}$. Then, the input is given importance weights for each channel, and long-term features ${\text{Z}}_{\text{N}}^{\text{L}}$ and short-term features $Z_{M}^{S}$ are extracted from the sEMG window. The temporal window set *D* consists of m windows; the *i* window is denoted by *X*_*i*_ ∈ ℝ*^HxW^*(1 ≤ *i* ≤ *M*), and the gesture label is denoted by *y_i_*. *H* is the number of EMG signal channels, and *W* is the number of sampling points per window.

Feature cross-attention module: This module receives the long-term features ${\text{Z}}_{\text{N}}^{\text{L}}$ and short-term features $Z_{M}^{S}$ extracted by the long short-term encoder. The long-term features and short-term features are cross-learned using scaled dot-product attention, and the cross-learned long-term features ${\text{Z}}_{\text{N}}^{L^{\prime }}$ and short-term features $Z_{M}^{S^{\prime }}$ are output.

Gesture classification module: This module receives the long-term features ${\text{Z}}_{\text{N}}^{L^{\prime }}$ and short-term features $Z_{M}^{S^{\prime }}$ after cross-learning, calculates the gesture category probabilities corresponding to the long short-term features, fuses the gesture probabilities for decision-level fusion and finally outputs the gesture categories.

### 3.1. Long short-term encoder

This module mainly consists of three parts: sEMG channel attention, linear projection, and the long/short-term sub encoder.

#### 3.1.1. Surface electromyography channel attention (ECA)

It is commonly accepted in medical statistics that sEMG signals from one muscle are statistically independent of those from neighboring muscles (Naik et al., 2007) and that specific muscles play more critical roles in certain hand movements (Huang et al., 2009). However, most of the previous methods extracted correlations between channels and gestures by constructing multistream inputs with channel decomposition signals. As an MSCNN (Wei et al., 2019) assigns network input streams to each channel and fuses them, the computational effort increases exponentially when the number of channels is high. Therefore, to reduce the computational effort, we propose modularized myoelectric channel attention based on scaled dot-product attention to perform correlation extraction of channels and gestures and dynamically adjust the channel weights according to the gestures, increasing the channel weights with strong correlations and decreasing the channel weights with weak correlations.

First, the sEMG window X_i_ converts each channel into K and Q. Then, we calculate the correlations between channels using scaled point multiplier attention and output the sEMG window with channel weights P_i_, as shown in Equation 2.


(2) $$P_{\text{i}}=X_{i}+S\,o\,f\,t\,m\,a\,x\,\left(\frac{A\,v\,g\,p\,o\,o\,l\,i\,n\,g\,\left(Q\right)\times A\,v\,g\,p\,o\,o\,l\,i\,n\,g\,\left(K\right)}{\sqrt{d_{k}}}\right)\,X_{i}$$

where *d*_*k*_ is the vector dimension, Avgpooling is the mean pooling layer, X_i_ is the raw EMG window, and sotfmax is a normalized exponential function.

#### 3.1.2. Linear projection

To construct long-term and short-term branches for multiscale feature extraction, this module slices *P_i_* into long-term and short-term segments, respectively, and performs linear projection into long-term tokens $p_{i}^{L}$ and short-term tokens $p_{i}^{S}$. The final construction forms the set of long-term branching tokens $z_{0}^{L}$. As in Equation 3, the set of short-term branching tokens $z_{0}^{S}$ is described in Equation 4.


(3) $$Z_{0}^{S}=\left[p_{\text{cls}}^{S};{\text{p}}_{1}^{S}\,E^{S};{\text{p}}_{2}^{\text{S}}\,E^{S};\ldots ;{\text{p}}_{S}^{N_{S}}\,E^{S}\right]+E_{\text{pos}}^{S}$$

(4) $$Z_{0}^{L}=\left[p_{\text{cls}}^{L};{\text{p}}_{1}^{{}_{L}}\,E^{L};{\text{p}}_{2}^{{}_{L}}\,E^{L};\ldots ;{\text{p}}_{N_{L}}^{{}_{L}}\,E^{L}\right]+E_{\text{pos}}^{L}$$

where *E^S^* and *E^L^* are linear projection matrices, $p_{1}^{s}\,p_{2}^{s}\,\ldots .p_{N_{S}}^{s}$ are short-term tokens whose sizes are set to *H* × *S*_*Short*_, $p_{1}^{L}\,p_{2}^{L}\,\ldots \,p_{N_{L}}^{L}$ are long-term tokens whose sizes are set to *H* × *S*_*Long*_, $P_{\mathrm{cls}}^{S}$ and $P_{\mathrm{cls}}^{L}$ are the classification tokens of the short-term branch and long-term branch, and $E_{\mathrm{pos}}^{S}$ and $E_{\mathrm{pos}}^{L}$ are the position embeddings of the short-term branch and long-term branch, respectively.

#### 3.1.3. Multihead reattention (MHRA)

The long-/short-term subencoder mainly consists of multihead reattention (MHRA) and a multilayer perceptron (MLP). MHRA is the contribution of this module. MHRA collects complementary information about the interactions between multiple attentions by adding a transformation matrix θ ∈ ℝ^*head*×*head*^. MHRA enables individual heads to observe the characteristics of the signal from different angles, effectively solving the attentional collapse problem (Zhou et al., 2021), where head is the number of MHRA output heads.

This module extracts the long- and short-term features from the set of long-term branch tokens $z_{0}^{L}$ and short-term branch tokens $z_{0}^{S}$ by MHRA and the MLP, respectively. The specific steps of the module are as follows.

First, we compute the interpatch attention information by transforming each patch in the output $z_{0}^{S}$ or $z_{0}^{L}$ of the linear projection module into QKV, which is fed into the respective branch’s encoder.


(5) $$R\,e-A\,t\,t\,e\,n\,t\,i\,o\,n\,\left(Q,K,V\right)=N\,\mathrm{orm}\,\left(\theta^{\text{T}}\,\left(S\,o\,f\,t\,\max\left(Q\,K^{T}/\sqrt{d_{\text{k}}}\right)\right)\right)\,V$$

where *Norm* is the layer norm normalization function, *Q*, *K*, and *V* are the query, key and value for the short-term branch, respectively, and *d_k_* is the vector dimension.

Next, the reattention information from the MHRA module is input to the MLP module, and the MHRA and MLP modules are connected by means of residuals. Finally, the short-term sequence characteristic of the short-term branch output is $Z_{M}^{S}$, as in Equation 6, and similarly, the long-term sequence characteristic $Z_{N}^{L}$ can be obtained with Equation 7.


(6) $$Z_{M}^{S}=\left[z_{\text{cls}}^{\text{S}};{\text{z}}_{1}^{S};\ldots ;z_{N_{S}}^{S}\right]$$

(7) $$Z_{N}^{L}=\left[z_{\text{cls}}^{L};{\text{z}}_{1}^{L};\ldots ;z_{N_{L}}^{L}\right]$$

where ${\text{z}}_{\text{cls}}^{\text{S}}$ and ${\text{z}}_{\text{cls}}^{L}$ are the classification tokens on the short- and long-term features, respectively, ${\text{z}}_{1}^{S}\,\ldots \,z_{N_{S}}^{S}$ are the patch tokens of the short-term features; *N*_*S*_ is the number of short-term patch tokens; ${\text{z}}_{1}^{L}\,\ldots \,z_{N_{L}}^{L}$ are the patch tokens of the long-term features; and *N*_*L*_ is the number of long-term patch tokens.

We stack the long- and short-term sub-encoders, M and N, respectively, to construct the deep network and extract deep features.

### 3.2. Feature cross-attention (FCA)

In the fields of image classification and object detection, a large number of researchers have proposed improved ideas for feature fusion methods (Yu et al., 2020; Zheng et al., 2020), such as feature pyramid networks (FPNs) (Lin et al., 2017), ResNet (He et al., 2016) and adaptive spatial feature fusion (ASFF) (Liu et al., 2019). The above research proved that setting up an appropriate feature fusion strategy is beneficial for improving accuracy. However, the current fusion methods are designed based on the feature maps extracted by convolutional neural networks and are not applicable to the vector features extracted by the transformer model. Therefore, we propose the feature cross-attention module (FCA) to cross-learn the classification token and patch tokens of two branches, which achieves the efficient fusion of long- and short-term features with less computational effort.

**FIGURE 4:**
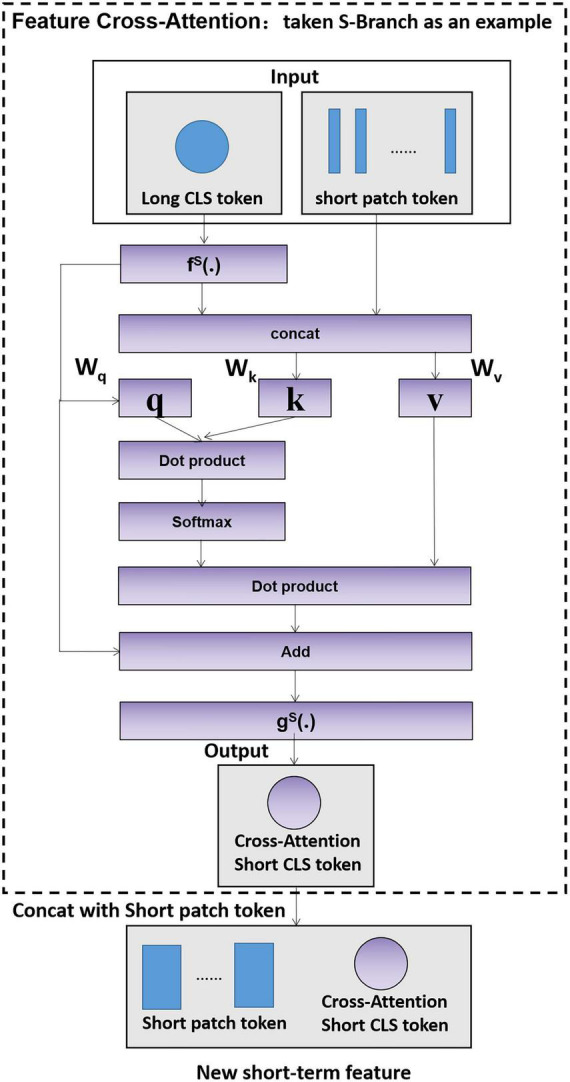
Feature cross-attention module.

Taking the short-term branch as an example, the feature cross-attention module is specified in Figure 4.

First, the short-term feature classification token (CLS token) and the long-term feature patch tokens are aligned and stitched together as in Equations 8, 9:


(8) $${\text{z}}_{\text{cls}}^{S^{\prime }}={\text{f}}^{\text{S}}\,\left({\text{z}}_{\text{cls}}^{\text{S}}\right)$$

(9) $${\text{z}}^{S^{\prime }}=\text{Concat}\,\left({\text{z}}_{\text{cls}}^{S^{\prime }},z_{1}^{L},\ldots ,z_{N_{L}}^{L}\right)$$

where f^S^(⋅) is the feature alignment function and Concat is the splicing operation.

Second, the FCA input ${\text{z}}_{\text{cls}}^{S^{\prime }}$ is cross-learned with z^*S′*^ as in Equations 10–13.


(10) $$F\,C\,A\,\left({\text{z}}_{\text{cls}}^{S^{\prime }},{\text{z}}^{S^{\prime }}\right)=\text{soft}\,\max\left(\frac{q\,k^{T}}{\sqrt{d}}\right)\,v$$

where q and k are the query and key of the short-term features, d is the long-term patch token dimension, and Softmax is the normalized exponential function.

Finally, the feature cross-attention is extended to multiple heads, which is denoted as multihead feature cross-attention (MFCA); the multihead features are aligned backward, and their output dimensions are kept consistent with the short-term feature classification token to obtain the short-term feature classification token $Z_{\text{cls}}^{S^{\prime \prime }}$ after cross-learning, as in Equation 11.


(11) $${\text{z}}_{\text{cls}}^{S^{\prime \prime }}=g^{S}\,\left({\text{z}}_{\text{cls}}^{S^{\prime }}+F\,C\,A\,\left({\text{z}}_{\text{cls}}^{S^{\prime }},{\text{z}}^{S^{\prime }}\right)\right)$$

where g^S^(⋅) is the reverse alignment function and ${\text{z}}_{\text{cls}}^{S^{\prime }}$is the classification token before reverse alignment.

At this point, the short-term feature after cross-learning is${}_{Z_{M}^{S^{\prime }}}$, as in Equation 12, and similarly, the long-term feature after cross-learning is $Z_{N}^{L^{\prime }}$, as in Equation 13.


(12) $$Z_{M}^{S^{\prime }}=\left[z_{\text{cls}}^{S^{\prime \prime }};{\text{z}}_{1}^{S};\ldots ;z_{N_{S}}^{S}\right]$$

(13) $$Z_{N}^{L^{\prime }}=\left[z_{\text{cls}}^{L^{\prime \prime }};{\text{z}}_{1}^{L};\ldots ;z_{N_{L}}^{L}\right]$$

Since the short-term feature classification token learns the abstract information of the branches, interacting with the patch tokens at the other branches helps to include information at a different scale. The fused long-term and short-term features $Z_{M}^{S^{\prime }}$ and $Z_{N}^{L^{\prime }}$ are output to the gesture classification model.

### 3.3. Gesture classification module

The short-term feature ${\text{z}}_{\text{cls}}^{S^{\prime \prime }}$ and long-term feature ${\text{z}}_{\text{cls}}^{L^{\prime \prime }}$ classification tokens are obtained, and the sum of the gesture scores of each branch is output to obtain the gesture category.


(14) $$\text{gestures}=L\,L\,\left(L\,a\,y\,e\,r\,N\,o\,r\,m\,\left({\text{z}}_{\text{cls}}^{S^{\prime \prime }}\right)\right)+L\,L\,\left(L\,a\,y\,e\,r\,N\,o\,r\,m\,\left({\text{z}}_{\text{cls}}^{L^{\prime \prime }}\right)\right)$$

## 4. Experiments and results

Our experiments employed a deep learning framework on a computer platform for model training and testing. The computer hardware configuration used was an Intel Core i7-8700K CPU processor (32 GB RAM) and a GeForce GTX 3090 GPU (24 GB RAM). The operating system was Ubuntu 18.04.4LTS, and network models were constructed, trained, and validated using the Python 3.6.5 programming language under the PyTorch 1.8.0 deep learning framework. The cross-entropy loss was used to measure classification performance.

### 4.1. LST-EMG-Net model training parameter setting

We evaluated different variants of the LST-EMG-Net architecture. For all model variants, we used the Adam optimizer to set the parameters to 0.9, 0.999, and the learning rate was corrected using StepLR, with the step size set to 3 and gamma set to 0.5. We set the initial learning rate to 6e-4 with a batch size of 512. The short-term patch length and long-term patch length were dynamically set according to the sEMG window length used for the dataset. For the short-term branch, the short-term subencoder depth was set to 1 (i.e., *M* = 1), and the number of short-term subencoder heads was set to eight. For the long-term branch, the long-term subencoder depth was set to 2 (i.e., *N* = 2), and the number of long-term subencoder heads was set to eight. The feature *cross-attention depth* = 4 (i.e., *L* = 4), and the number of feature *cross-attention heads* = 8. All models were trained under these parameters until convergence.

### 4.2. Ablation experiments

In this article, we evaluate the LST-EMG-Net model on three datasets and describe the ablation experiments of the LST-EMG-Net model. This ablation experiment used the ViT model extended to dual streams as the baseline and added the gesture recognition effects of FCA, ECA, MHRA, and SA. The baseline model framework was used to remove the yellow module in Figure 3. We recorded the average accuracy of all subjects on each dataset to form Table 1.

**TABLE 1:** Hand gesture recognition accuracy achieved on each dataset in the ablation experiments.

Model name	FCA	ECA	MHRA	SA	DB2 exercise B	DB5 exercise C	CapgMyo DB-c
Baseline					76.25%	77.17%	91.60%
Model 1	√				78.13% (+1.88%)	83.00% (+5.83%)	92.24% (+0.64%)
Model 2	√	√			79.51% (+1.38%)	85.53% (+2.53%)	96.53% (+4.29%)
Model 3	√	√	√		80.62% (+1.11%)	86.96% (+1.43%)	**98.95% (+2.32%)**
Model 4	√	√	√	√	**81.47% (+0.85%)**	**88.24% (+1.28%)**	98.80% (−0.15%)

The bold value means the best recognition accuracy.

LST-EMG-Net shows the best model results obtained by simultaneously adding FCA, ECA, MHRA, and SA. Model 1 improves the average recognition accuracy by 2.78% compared to the baseline. This demonstrates that dual-stream information fusion helps improve accuracy. Model 2 improved the average recognition accuracy by 2.73% on the three datasets compared to Model 1, with a 4.29% improvement on the CapgMyo DB-c high-density dataset. Because of the high number of channels of the sEMG acquisition device in this dataset, 128 channels, and the rich muscle activity information between channels, the ECA effect is evident in this dataset. Model 3 improved the average recognition accuracy by 1.62% compared with model 2. Because the amount of sEMG data in this experiment was smaller and the number of required network layers was relatively shallow, with the addition of MHRA, the network may perform better on more extensive sEMG data. Model 4 achieves an average recognition accuracy improvement of 0.66% compared to model 3. However, on the CapgMyo DB-c high-density dataset, the original signal size reached saturation due to the high sampling rate and the number of channels in this dataset. Therefore, compared to model 3, the model accuracy stabilized.

### 4.3. Comparison experiment

We compare the proposed LST-EMG-Net with the existing MSCNN of the multistream CNN (Wei et al., 2019), the bidirectional temporal convolutional network (BiTCN) (Chen et al., 2020), and TEMG based on the vision transformer (ViT) (Siddhad et al., 2022) on the above three EMG datasets.

(1) LST-EMG-Net’s accuracy and inference time: The performance is shown in Table 2.

**TABLE 2:** Accuracies and inference times of LST-EMG-Net and the comparison algorithms.

Dataset	Model name	Accuracy	Inference time
DB2 exercise B	MSCNN	71.89%	5.60 ms
	BiTCN	65.79%	5.75 ms
	TEMG	78.77%	1.09 ms
	LSTEMGNet [ours]	**81.47%**	6.47 ms
DB5 exercise C	MSCNN	79.14%	7.27 ms
	BiTCN	83.75%	7.29 ms
	TEMG	68.18%	1.18 ms
	LSTEMGNet [ours]	**88.24%**	6.36 ms
CapgMyo DB-C	MSCNN	86.67%	7.78 ms
	BiTCN	98.38%	7.30 ms
	TEMG	92.90%	1.12 ms
	LSTEMGNet [ours]	**98.80%**	6.32 ms

The bold value means the best recognition accuracy.

From Table 2, we can see that our method reaches the optimum results on the three datasets of DB2 Exercise B, DB5 Exercise C, and CapgMyo DB-C, and the accuracy is improved by 2.70, 4.49, and 0.42%, respectively, compared to the optimum comparison methods.

Regarding the recognition time aspect, we can also see from Table 2 that LST-EMG-Net not only has higher recognition accuracy but also outperforms the CNN-based MSCNN model and the RNN-based BiTCN model in terms of inference time. Both LST-EMG-Net and TEMG are designed based on the transformer model, but the difference is that LST-EMG-Net extends the transformer to a dual-flow structure. Compared with the single-stream structure of TEMG, the average recognition accuracy of LST-EMG-Net is 9.5% higher on the three datasets, which demonstrates improved recognition accuracy and stability. Furthermore, the dual-stream structure of LST-EMG-Net increases the computational and parametric quantities of the model to a certain extent. On average, it is 5.25 ms slower than TEMG, but both can meet the requirements of real-time recognition.

(2) LST-EMG-Net’s stability: To verify the stability of LST-EMG-Net in recognizing various types of gestures, we compare the fluctuation of the recognition accuracy of the method in this article with that of MSCNN, BiTCN, and TEMG. We choose the standard deviation (STD) as an indicator to measure the fluctuation of each gesture between subjects. Taking gesture one as an example, the fluctuation value is calculated as follows in Equation 15.

(15) $$\mathrm{G1}=\sqrt{\frac{\sum_{i=1}^{n}a\,c\,c_{i}-\bar{a\,c\,c}}{n}}$$

where i is the subject number, acc_i_ denotes the i-th subject gesture one accuracy, $\bar{a\,c\,c}$ is the average gesture pne accuracy, and n is the number of subjects. A smaller fluctuation value means that the gesture recognition is more stable, and we calculate the average fluctuation value of each gesture in the three datasets, as shown in Table 3.

**TABLE 3:** Average fluctuation values of LST-EMG-Net and the comparison algorithms.

Model name	DB2 exercise B	DB5 exercise C	CapgMyo DB-C
MSCNN	0.1795	0.1835	0.1252
BiTCN	0.2232	0.1324	0.0396
TEMG	0.1197	0.1392	0.1045
LST-EMG-Net [ours]	**0.1181**	**0.1098**	**0.0179**

The bold value means the highest recognition stability.

The experimental results in Table 3 show that the average fluctuation value of the proposed LST-EMG-Net is low for all kinds of gestures. It is suitable for recognition tasks because it learns the information of EMG sampling points with different timing lengths, thus maintaining a relatively stable and high recognition rate for gestures of different complexity.

## 5. Conclusion

Current research gives little attention to the problem of matching the amount of information in features with the amount of information needed to recognize gestures. Here, we propose the LST-EMG-Net-based sEMG gesture recognition method to address the above problems; it is mainly composed of a long short-term encoder and a feature cross-attention module. Our method maintains a high level of accuracy for all types of gesture recognition in both sparse EMG datasets and high-density sEMG datasets. It improves the stability of gesture recognition compared to other network structures.

Our LST-EMG-Net framework can be applied well to recognize various types of gestures by subjects. Nevertheless, due to the individual variability among subjects, LST-EMG-Net is difficult to apply to the intersubject recognition of gestures and has a high burden of use for new subjects, which needs further study in clinical applications. In the future, we will improve the LST-EMG-Net framework to achieve intersubject gesture recognition for controlling exoskeletons or other rehabilitation devices for post-surgical rehabilitation of stroke patients.

## Data availability statement

Publicly available datasets were analyzed in this study. This data can be found here: http://ninapro.hevs.ch/node/7.

## Ethics statement

Written informed consent was obtained from the individual(s) for the publication of any potentially identifiable images or data included in this article.

## Author contributions

WZ and TZ conceived the ideas and designed the methodology, implemented the technical pipeline, conducted the experiments, and analyzed the results. JZ and YW provided the dataset for the experiments. All authors discussed the manuscript, wrote the manuscript, and gave final approval for its publication.
